# Death to the Coral Reefs and More Money for Everybody Else

**DOI:** 10.1100/tsw.2000.10

**Published:** 2000-10-27

## Abstract

It was a bad week for the residents of the world’s rapidly disappearing coral reefs, says Science in its lead story. But it was a good week to be a mathematician. NSF director Rita Colwell proposed tripling their research budget, reports Nature’s lead story.

You don't need an advanced degree in math to understand the impact of *Science*'s lead story: the coral reefs are in trouble. And it's probably our fault. In addition to local environmental insults like pollution and destructive fishing practices, the world's reefs received a big ecological slap in the face from unusually warm currents carried by the 1997-8 El Niño event thought to be the product of global warming, reports *Science*. The reefs were already in bad shape. Prior to 1998, an estimated 11% of the world's known reefs had been destroyed by human activities, according to a report presented last week at a conference in Indonesia by the Global Coral Reef Monitoring Network. But the warm currents took the situation from bad to worse. Barely 1 year later, the warm waters of El Niño had "severely damaged" another 16%. That's a grand total of 27% of the world's coral reefs, folks, TWENTY-SEVEN PERCENT! On happier note, some reefs have recovered more rapidly than predicted, says *Science*, and increasingly conservation-minded tropical nations are becoming reluctant to bite the undersea hand that feeds them.

*Nature* also covers the report in substantially the same fashion, but they dropped the story to slot four. Which raises the question: since when is the destruction of 27% of the world's coral reefs the fourth most important story of the week?

In their lead story, *Nature* reports that Rita Colwell is again doing what scientists think she does best: giving out money. This week's beneficiaries are the mathematicians, who could receive a funding windfall of as much as $500 million per year by 2007. Colwell argues that despite the central role of math in the big three "technologies" — info, bio, and nano — the supply of mathematicians is drying up. Instead of toiling away in graduate school on grants that average $10,000 per year and annual stipends of $16,800, the best and brightest math minds of the U.S., Europe, and Asia are heading straight into the lucrative field of computer programming. Skeptical mathematicians will applaud the increase, "if it happens," reports *Nature*.

In a related story, *Science* reports that the U.S. Congress approved generous increases in next year's budget for NASA and the NSF. The projected overall budget surplus and election-year largesse combined to boost the 2001 NSF budget by 17% to $4.42 billion and raised the NASA budget 6% to $14.2 billion. *Science* reports that instead of splitting the difference between their initial budget requests, the Congress and White House "compromised" on a total for both NSF and NASA that exceeded the first offers from either group. Colwell was pleased, says *Science*, quipping, "I really like Congress's math this year." *Nature* did not cover the announcement.

Finally, both magazines report the discovery of 4 new moons orbiting Saturn, which now has more satellites than any other planet. With a total of 22, Saturn has 1 more moon than Uranus, the previous champion.
